# Serum pro-inflammatory biomarkers associated with improvement in quality of life in pulmonary tuberculosis

**DOI:** 10.3389/fimmu.2023.1241121

**Published:** 2023-09-11

**Authors:** Laura E. Carreto-Binaghi, Luis Gustavo Sartillo-Mendoza, Marcela Muñoz-Torrico, Silvia Guzmán-Beltrán, Claudia Carranza, Martha Torres, Yolanda González, Esmeralda Juárez

**Affiliations:** ^1^ Laboratorio de Inmunobiología de la Tuberculosis, Instituto Nacional de Enfermedades Respiratorias Ismael Cosío Villegas, Mexico, Mexico; ^2^ Departamento de Investigación en Microbiología, Instituto Nacional de Enfermedades Respiratorias Ismael Cosío Villegas, Mexico, Mexico; ^3^ Facultad de Medicina, Benemérita Universidad Autónoma de Puebla (BUAP), Puebla, Mexico; ^4^ Becario de la Dirección General de Calidad y Educación en Salud, Secretaría de Salud, Mexico, Mexico; ^5^ Clínica de Tuberculosis, Instituto Nacional de Enfermedades Respiratorias Ismael Cosío Villegas, Mexico, Mexico

**Keywords:** tuberculosis, quality of life, eicosanoids, redox indicators, inflammation-associated lung injury

## Abstract

**Introduction:**

Pulmonary dysfunction is an underestimated complication in tuberculosis (TB) infection, affecting quality of life (QoL). Although respiratory function tests objectively reflect lung disturbances in a specific moment, predictors of illness severity at the time of diagnosis are still lacking.

**Methods:**

We measured serum pro-inflammatory cytokines (TNF-α and IL-8), eicosanoids (PGE2, LTB4, RvD1, Mar1, and LXA4), a marker of tissue damage (cell-free nucleosomes), and indicators of redox status (malonaldehyde, 8-isoprostane, total oxidants, and antioxidants), as well as a score of radiological abnormalities (SRA) and a QoL questionnaire, in 25 patients with pulmonary TB at the time of diagnosis (t0) and two months after the initiation of treatment (t2).

**Results:**

We found higher antioxidant levels in the patients with the worst QoL at t0, and all the indicators of the prooxidant state were significantly reduced at t2, while the total antioxidant levels increased. LTB4, a pro-inflammatory eicosanoid, was diminished at t2, while all the pro-resolutory lipids decreased substantially. Significant correlations between the SRA and the QoL scores were observed, the latter showing a substantial reduction at t2, ranking it as a reliable tool for monitoring disease evolution during TB treatment.

**Discussion:**

These results suggest that evaluating a combination of these markers might be a valuable predictor of QoL improvement and a treatment response indicator; in particular, the oxidation metabolites and eicosanoid ratios could also be proposed as a future target for adjuvant therapies to reduce inflammation-associated lung injury in TB disease.

## 1 Introduction

Before the COVID-19 pandemic, the World Health Organization recognized tuberculosis among the top 10 causes of death in low and lower-middle-income countries (1). Despite the efforts to develop a global strategy against tuberculosis, this respiratory infection remains a public health crisis (2). Although effective anti-tuberculosis treatment is available, social determinants must be addressed to grant all patients access to medicines (2). Calculation of TB-related disease burden should consider the likelihood of a residual pulmonary disability.

One of the critical challenges in tuberculosis is the timely detection of pulmonary dysfunction, which might impact the patient’s quality of life after diagnosis. Pulmonary function tests, such as spirometry, are standard evaluation methods to quantify the precise air volume within the lungs, reflecting lung capacity and function, allowing long-term monitoring for patients (3). However, performing spirometry requires specific technical instruments, and results depend on operator-dependent ability, expertise, and the patient’s cooperation during the test (3). Pulmonary function in patients with pulmonary TB may also be assessed indirectly by health-related quality of life (HRQoL) questionnaires, such as the St. George Respiratory Questionnaire (SGRQ) (4, 5). Higher SGRQ scores at treatment initiation have shown to predict poor treatment outcomes in Indian TB patients, and their increment during follow-up has been related to the risk of TB recurrence (6); an improvement in SGRQ scores has also been determined in patients undergoing therapeutic surgery for pulmonary TB (7), straightening out the consistency of the questionnaire.

Recent research points toward using biomarkers to improve diagnostic accuracy in patients with lung dysfunction. For instance, CT scans and other imaging technologies often identify biomarkers extracted from 3D lung segmentations to follow the tuberculosis progression (8); also, integrating metabolomics and transcriptomics has improved biomarker discovery, leading to earlier detection and treatment (9). However, these techniques can be expensive and time-consuming, thus urging the need for non-invasive procedures to evaluate low-cost biomarkers which can predict disease severity and outcomes.

The severity of pulmonary TB has been associated with different environmental factors: seasonality, latitude, photoperiod, radiation (10), and also genetic variants related to vascular biology, inflammation (11), and oxidative stress (12). Compared with healthy controls, TB patients show severe oxidative stress through higher levels of lipid peroxidation products, free radical activity, and lower total antioxidant capacity (13, 14). The interplay between oxidative stress, systemic inflammation, and tissue remodeling in TB disease suggests that improving host antioxidant status may be a reasonable strategy to ameliorate tissue damage after TB treatment (15).

Because of their role in modulating the immune response during TB infection, several cytokines have also been proposed as potential biomarkers to predict disease severity. For instance, IL-3, IL-12p40, LIF (leukemia inhibitory factor), IFN-α2, IL-2ra, IL-13, β-NGF (nerve growth factor), SCF (stem cell factor), TNF-β, TRAIL (tumor necrosis factor-related apoptosis-inducing ligand), IL-2, IFN-γ, IP-10, and MIG (membrane-bound immunoglobulin) were significantly higher in both active and latent TB compared to other respiratory infections, while MIF (macrophage migration inhibitory factor) was significantly lower in active TB patients only (16). Likewise, IL-17F, MIP-3α, IL-13, IL-17A, IL-5, IL-9, IL-1β, IL-2, and IFN-γ identify TB and distinguish between latent and active stages (17). In animal TB models, severe TB infection induces up-regulation of genes involved in Th1 and Th17 responses, and tissue overexpression of IL-22, MIP-1α, CCL27, IP-10, CCR4, CCR5, CXCR3, PD-1, PDL-2, IL-3, IFN-β, TIM-1, and TLR-2 associated with low TB-specific cellular responses (18).

Nevertheless, follow-up of these potential biomarkers is not feasible in every socioeconomic condition. More conventionally available reagents, such as C reactive protein (CRP), IL-6, IP-10, and TNF-α exhibit a promising position for the TB treatment monitoring (19), while TSP4 (thrombospondin 4), TIMP-2 (tissue inhibitor of metalloproteinase-2), SEPR (fibroblast activation protein α), MRC-2 (mannose receptor C type 2), antithrombin III, serum amyloid A, CRP, phospholipase A2, hepcidin, and LPS-binding protein exhibit significant expression differences during the intensive phase of TB therapy (20).

Eicosanoid modulation is a potential target for therapeutic intervention in TB due to the role of these molecules during TB infection. *Mycobacterium tuberculosis* inhibits apoptosis and promotes necrotic cell death by disrupting prostaglandin E2 (PGE2) production, which delays T-cell priming and favors mycobacterial immune evasion (21). Moreover, eicosanoid levels are increased in individuals with TB and TB-diabetes comorbidity, where eicosanoid ratios can reflect disease severity and extent (22). The balance between lipoxin A4 (LXA4) and PGE2 is critical for controlling TB immunopathology, and PGE2 signaling via EP2 receptor is a host-protective pathway for *M. tuberculosis* infection (23).

In this study, we evaluated the oxidative response and lipid mediators and their interrelationships as immunological biomarkers, combined with a radiological score and an HRQoL questionnaire, seeking a functional clinical tool to predict severity in pulmonary TB patients.

## 2 Materials and methods

### 2.1 Study settings

This study was conducted at the Instituto Nacional de Enfermedades Respiratorias Ismael Cosío Villegas (INER), a third-level institution providing specialized care for tuberculosis and other respiratory diseases. The institution offers immediate care for patients’ needs and then refers them to first-level attention centers to supervise their direct observed therapy. Upon request, some patients may be called back for a subsequent visit. The Institutional Review Board approved the study, approval number C53-17.

### 2.2 Study participants

Twenty- five persons aged 18-65 with pulmonary TB who requested attention at INER between February 2022 and March 2023 and had been on treatment for no more than two weeks were eligible to participate in the study. Patients with chronic inflammatory diseases, asthma, chronic obstructive pulmonary disease (COPD), and cancer, and those pregnant or living with HIV were ineligible. All patients were diagnosed with active pulmonary TB after a sputum smear-positive test or Xpert^®^ MTB/RIF assay, further confirmed by *M. tuberculosis* culture.

The subjects undertook radiological and clinical examinations. Disease severity was evaluated considering the smear report, laterality of the lung lesions, presence of cavities, and the score of radiographical abnormalities (SRA), as previously reported (24, 25). The SRA evaluates the presence, distribution, and extent of consolidation, fibrosis, lung distortion, bronchiectasis, and parenchymal abnormalities by quadrants in the chest X-ray. The score denotes the percentage of lung parenchyma involvement; the maximum score was 20 points, and a specialized pulmonologist performed the measurement.

All participants gave written informed consent and provided a blood sample at recruitment (t0). Eleven patients were willing to give a second sample two months after the first visit (t2), and three patients provided a third sample six months after the first visit (t6). Serum obtained by centrifugation was cryopreserved at -80°C until use.

### 2.3 St. George’s respiratory questionnaire

All participants completed the HRQoL questionnaire at each visit. The SGRQ weighted responses produce a score for each of three sections (symptoms, activities, and impact), which are then combined to obtain a total score. The symptoms section assesses the frequency and severity of respiratory symptoms; the activities section estimates the weakening of mobility or physical activity; the impact component evaluates the social and psychological effects of pulmonary dysfunction. The SGRQ is scaled from 0 to 100 (best and worst quality of life, respectively); we used the SGRQ scoring calculator app developed at the University Hospital of Copenhagen (26). For comparison and analysis, the total score was used; the impact score was used where indicated. To prevent bias from different levels of reading comprehension, the otherwise self-administered questionnaire was read to participants by trained physicians using the questionnaire manual. We used the Mexican Spanish version of the questionnaire (27). Subjects answered all questions. For comparison purposes, ten healthy individuals with normal chest X-ray images and laboratory results were included exclusively for SGRQ scoring.

### 2.4 Measurement of serum metabolites

#### 2.4.1 Eicosanoids

PGE2, LTB4, RvD1, Mar1, and LXA4 concentrations were quantified using commercial EIA kits (Cayman Chemical, Ann Harbour, MI, US). All assays were performed in duplicates according to manufacturer’s instructions. Serum samples underwent extraction using ethanol precipitation before analysis. Optical density was determined at 450 nm using a microplate reader (MultisKan Ascent, Agilent Technologies Inc., Santa Clara, CA, US).

#### 2.4.2 Cytokines

TNF-α was measured as previously reported (28); IL-8 quantification was measured by ELISA, according to the kit manufacturer’s instructions (Mabtech, Nacka Strand, SE).

#### 2.4.3 Nucleosomes

Cell-free nucleosomes were measured using the Cell Death Detection ELISA Plus kit (Roche Diagnostics, Indianapolis, IN, US), which detects DNA and histones for specific mono- and oligonucleosomes detection, following the manufacturers’ instructions. Results are reported as a percentage of the positive control included in the kit as reported elsewhere (25, 29).

#### 2.4.4 Lipid peroxidation indicators

Malonaldehyde (TBARS) and 8-isoprostane concentrations were quantified in duplicates using commercial kits, following the manufacturer’s instructions (Cayman Chemical).

#### 2.4.5 Total oxidants

Measurement was performed using the Total Oxidant Status (TOS) Colorimetric Assay Kit (Elabscience, Houston, TX, US). The detection principle is based on the ability of the contents of the sample to oxidize Fe2+ to Fe3+, which binds xylenol orange to produce a blue-purple complex. The color intensity was directly proportional to absorbance at 590 nm, proportional to the sample’s oxidative substances content.

#### 2.4.6 Total antioxidants

Measurement was performed using the Total Antioxidant Status (TAS) Colorimetric Assay Kit (Elabscience). Briefly, ABTS is oxidized to ABTS•+ (green), which can be reduced to a colorless ABTS in the presence of antioxidants. The TAS of the sample was calculated by measuring the absorbance of ABTS•+ at 660 nm. Trolox was used as a reference substance.

### 2.5 Statistical analysis

The baseline characteristics of the TB patients included in the analysis were presented as numbers and percentages or medians with range. These results are descriptive, and no comparison test was performed. Correlations were determined using Spearman correlation. A two-way analysis of variance with between-group comparisons was performed with a *post hoc* significance set at 0.05. Nonparametric test statistics were performed: Friedman’s followed by Dunn’s tests for related data and Kruskal-Wallis’ followed by Dunn’s test for unrelated data; for comparison between two variables, we used Wilcoxon’s rank sum or Mann-Whitney U, depending on the pairing. For comparisons at t0, t2, and t6, we first confirmed normality with Shapiro Wilk’s test; then we performed One-way ANOVA followed by Geisser-Greenhouse’s epsilon and Holm-Šídák’s multiple comparisons tests. Principal component analysis (PCA) was performed on multiple variables, and PC scores and loadings plots were displayed. All analyses used GraphPad Prism version 9.5.1 (GraphPad Software, La Jolla, CA, US).

## 3 Results

### 3.1 Characteristics of the patients

We included 25 patients with confirmed pulmonary TB; no sex predominance was observed in this group (**Table 1**). In 84% of cases, drug-susceptible *M. tuberculosis* was identified, and the patients received a WHO-recommended regimen (isoniazid, rifampicin, pyrazinamide, and ethambutol). Multidrug-resistant TB was found in three cases, and one patient had monorresistant TB; their individualized treatment was established by specialized pulmonologists.

**Table 1:** Demographic and clinical characteristics of the tuberculosis patients.

Characteristic	N=25
Women, n (%)	13 (52)
Age, years, median (range)	47 (19-61)
BMI kg/m^2^, median (range)	21.62 (14.86-42.69)
Treatment days, median (range)	1 (0-14)
Comorbidities:	
Diabetes, n (%)	9 (36)
Smoking, n (%)	4 (16)
Rheumatic diseases, n (%)	2 (8)
Hypertension, n (%)	1 (4)
Recent drug history*:	
NSAIDs, n (%)	7 (28)
Metformin, n (%)	4 (16)
Insulin, n (%)	3 (12)
Aspirin, n (%)	1 (4)
Sulfonylurea, n (%)	1 (4)
Antihypertensive medication, n (%)	1 (4)
Disease severity:	
Bilateral, n (%)	14 (56)
Cavities, n (%)	16 (64)
SRA, median (range)	10 (0-20)
SGRQ total score, median (range)	62 (5.92-97.67)
Smear grade (BK), n (%)	
0	11 (44)
1+	2 (8)
2+	3 (12)
3+	9 (36)

*Patients were asked about taking these drugs in the two weeks before blood sampling.

SRA: score of radiographic abnormalities, SGRQ: St. George’s Respiratory Questionnaire.

**Table 1** depicts certain features of disease severity, which were further analyzed for this study. Because diabetes, *M. tuberculosis* drug resistance, and smoking potentially affect our results, we performed formal hypothesis tests to assess whether the variables were associated with the outcome. We found that none of these variables affected the levels of the biomarkers (data not shown).

### 3.2 Exploring interrelationships between circulating resolution of inflammation lipids, pro-inflammatory cytokines, and redox biomarkers in pulmonary tuberculosis

**Figure 1:**
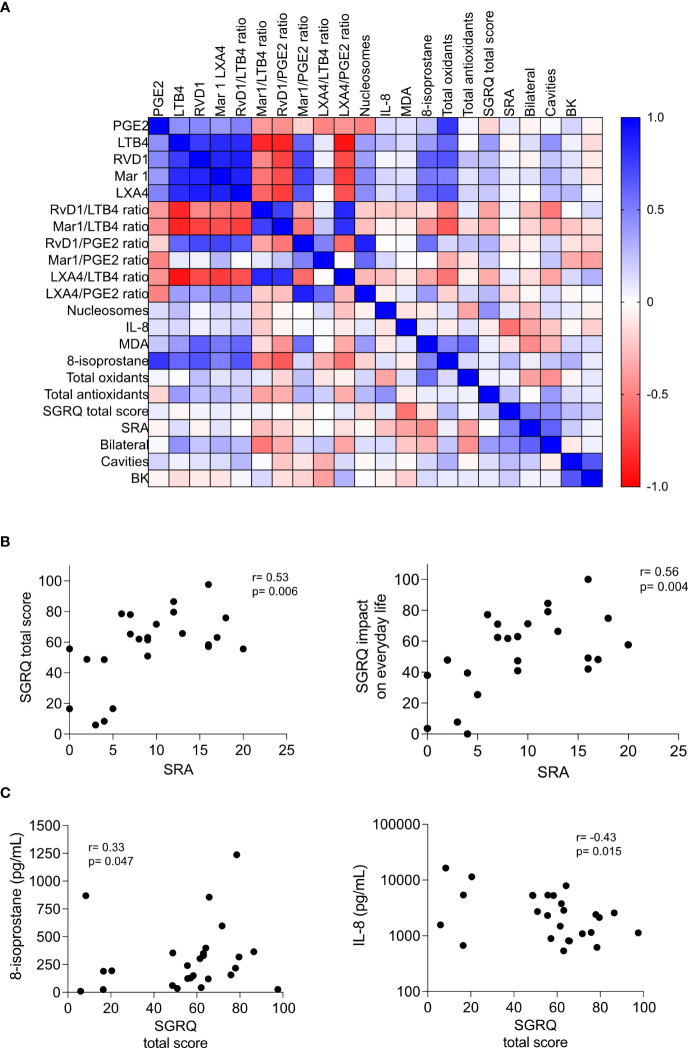
Associations between circulating biomarkers and disease severity in pulmonary tuberculosis. **(A)** Heatmap showing the correlation between various biomarkers, including lipid mediators, tissue damage markers, pro-inflammatory cytokines, redox state indicators, quality of life (QoL) indicators, and lung damage indicators in patients with pulmonary tuberculosis evaluated at the time of recruitment (t0); the Spearman’s Rho heatmap is depicted. **(B, C)** Correlation of the St. George’s Respiratory Questionnaire (SGRQ) scores with the damage of lung parenchyma measured by the Score of Radiologic Abnormalities (SRA) **(B)**, and 8-isoprostane and IL-8 **(C)**. The Spearman’s Rho and p values are depicted, n=25.

To identify the presence and strength of associations between variables, we calculated correlation coefficients for each pair of variables in Spearman’s correlation and visualized them in a matrix heatmap. We analyzed lipids involved in inflammation and its resolution, pro-inflammatory cytokines TNF-α and IL-8, cell-free nucleosomes, indicators of lipid peroxidation (MDA and 8-isoprostane), total oxidants and antioxidants, quality of life (QoL) SGRQ scores, and indicators of lung involvement such as the score of radiologic abnormalities (SRA), the laterality of the damage, and the smear grade (BK) obtained at the first visit of the patients to the clinic (t0). We included calculations for the pro-resolutory/pro-inflammatory lipids ratio, considering that eicosanoid effects may depend more on their relative contribution rather than on their absolute levels (30). The correlation matrix (**Figure 1A**) showed whether they were positively or negatively related or had no significant relationship: shades of blue represented positive correlations, while shades of red represented negative correlations, and the color intensity corresponds to the strength of the correlation.

We observed patterns and clusters in the heatmap. The lipids involved in the inflammation and its resolution were highly correlated with the lipid peroxidation markers, while the QoL and lung damage indicators did not correlate well with other variables. All Spearman’s Rho and p values can be found in **Supplementary Material 1**. Although QoL scores lacked correlation with the serum metabolites, we found significant correlations between SGRQ scores and the SRA, depicted in additional correlation graphs including 24 patients, as one of them did not have a corresponding chest X-ray (**Figures 1B, C**). These results suggest that the measurement of QoL indicators may help understand the connection between lung damage and the impact of the respiratory symptoms on everyday life. Variables that exhibited a strong correlation with each other merit further investigation.

### 3.3 Comparative analysis of serum metabolite levels concerning disease severity

Because correlations do not imply causation, and the heatmap may not adequately capture non-linear relationships, investigating their interrelationships is still crucial for understanding the connections between different circulating metabolites and lung damage. We divided the group according to various severity indicators, namely, the laterality of the lung damage, the presence of cavities, and the smear grade. In addition, we divided the group according to the SGRQ total score, using a cut-off of 23 points. Although people in good health, whose pulmonary functions are optimal, usually yield SGRQ total scores lower than 12 (4), we chose 23 as this fits the lowest values for Mexican COPD patients and would be suggestive of the most severe respiratory distress (31, 32). This last classification was challenging because our study group consisted of patients whose QoL was poor at baseline. Thus, the SGRQ total score classification grouped only four patients with scores lower than 23; the other categories produced more evenly distributed groups.

**Figure 2:**
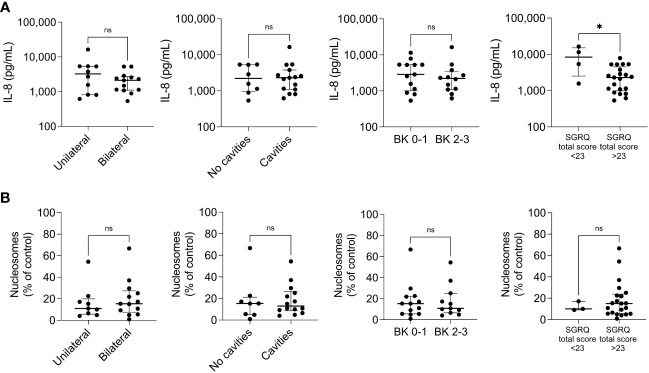
Pro-inflammatory mediators across disease severity indicators. The circulating levels of the cytokine IL-8 **(A)** and cell-free nucleosomes **(B)** were measured by ELISA in patients with pulmonary tuberculosis at the time of recruitment (t0), n=25. The patients were categorized into two groups according to the extent of lung damage (extreme left), the presence of cavities (center left), the smear grade (BK, center right), and the SGRQ total score (extreme right). Individual values with median and interquartile ranges are depicted; ns= not significant; * p<0.05, Mann-Whitney U test.

**Figure 3:**
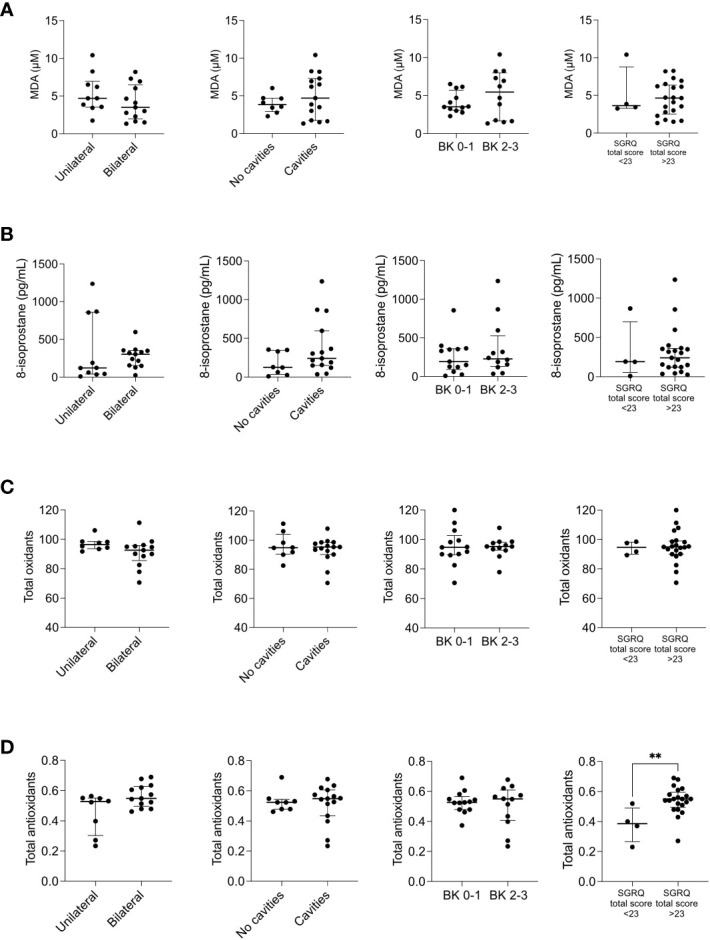
Redox state mediators across disease severity indicators. The circulating levels of malonaldehyde (MDA) **(A)**, 8-isoprostane **(B)**, total oxidants **(C)**, and total antioxidants **(D)** were measured by ELISA in patients with pulmonary tuberculosis at the time of recruitment (t0), n=25. The patients were categorized into two groups depending on the extent of lung damage (extreme left), the presence of cavities (center left), the smear grade (BK, center right), and the SGRQ total score (extreme right). Depicted are individual values with median and interquartile ranges. **p<0.01, Mann-Whitney U test.

While investigating the association of eicosanoids circulating levels with the severity of the disease, we found that LTB4 tended to be higher in those patients with bilateral lung involvement; however, none of the lipids were significantly associated with the severity of the disease (**Supplementary Figure 1**). Eicosanoid balance may be affected during tuberculosis infection, but the ratios of pro-resolutory/pro-inflammatory eicosanoids showed no association with disease severity (**Supplementary Figure 2**). When analyzing the cytokine results, two outcomes were surprising: the circulating TNF-α levels were undetectable (data not shown), and IL-8 levels were significantly lower in patients with a diminished QoL (**Figure 2A**). We also analyzed the cell-free nucleosomes, which have been proposed as tissue damage indicators and surrogates of neutrophil extracellular traps (NETs) (33) and expected to circulate in patients with severe systemic inflammation (34). However, we found no association with disease severity (**Figure 2B**). Similarly, we found that the levels of biomarkers related to the redox state were the same for all patients, regardless of the classification of disease severity (**Figures 3A-D**), except for the total antioxidant levels, which were higher in the patients with the worst QoL (**Figure 3D**).

**Figure 4:**
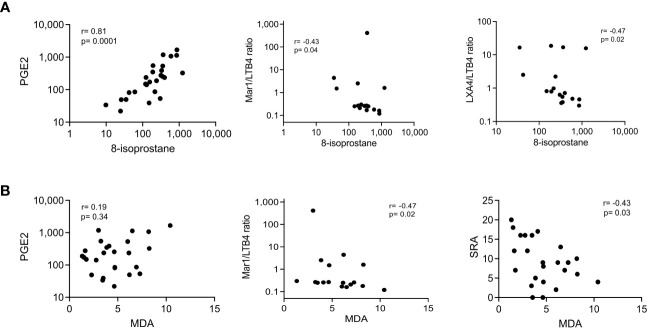
Correlation of lipid peroxidation markers with pro-inflammatory mediators and lung parenchyma damage. Serum biomarkers were measured by ELISA at the time of recruitment (t0); lung parenchyma damage was estimated by the Score of Radiologic Abnormalities (SRA). Correlation graphs of 8-isoprostane **(A)** and MDA **(B)** with PGE2 (left), Mar1/LTB4 ratio (center), and LXA4/LTB4 ratio or SRA (right) are displayed; the Spearman’s Rho and p values are depicted, n=25.

The group was highly homogeneous, which explains the inability to associate the circulating metabolites with the severity of the disease. Nonetheless, identifying common trends highlights interesting relationships or dependencies between variables. One of them was observed between pro-inflammatory lipids such as PGE2 and LTB4 ratios and lipid peroxidation indicators such as MDA and 8-isoprostane (**Figures 4A, B**). Because lipid peroxidation marker MDA inversely correlated with the SRA (**Figure 4B**, right), this relationship merited further investigation.

### 3.4 Dynamic changes in inflammatory and redox biomarkers during antituberculosis treatment: unexpected findings and alterations in pro-resolutory/pro-inflammatory ratios

**Figure 5:**
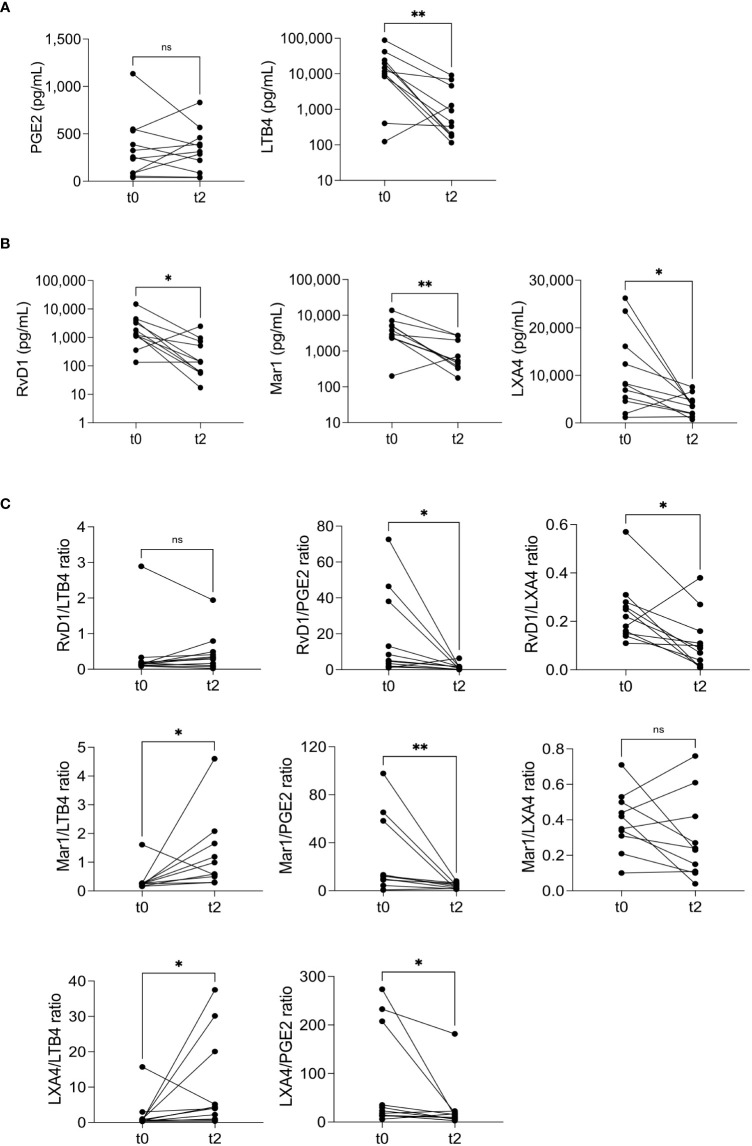
Eicosanoid modulation after two months of treatment. Pro-inflammatory **(A)** and pro-resolutory lipids **(B)**, and pro-resolutory to pro-inflammatory ratios **(C)** were measured at recruitment (t0) and two months after the first visit (t2). The graphs compare each individual’s before/after levels, n=11; ns= not significant, *p<0.05, **p<0.01, Wilcoxon’s Rank sum.

**Figure 6:**
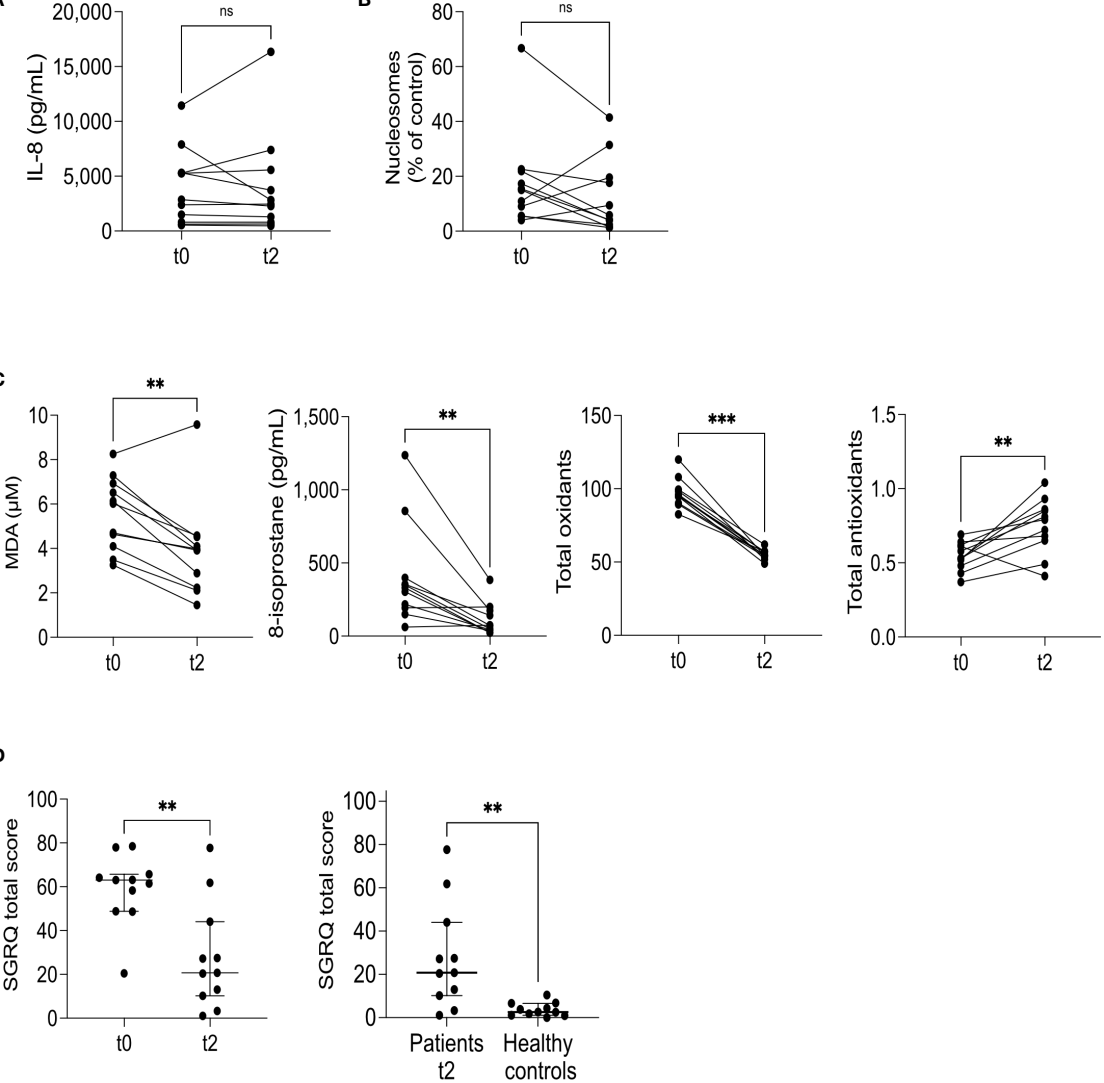
Cytokines and redox state mediators’ modulation at two months. IL-8 **(A)**, cell-free nucleosomes **(B)**, and redox state markers **(C)** were measured at recruitment (t0) and two months after the first visit (t2). The graphs compare each individual’s before/after levels, n=11; ns= not significant, **p<0.01, ***p<0.001, Wilcoxon’s Rank sum. The SGRQ total score was calculated at t0 and t2 (**D**, left), n=11, **p<0.01, Wilcoxon’s Rank sum. The SGRQ total score at t2, n=11, was compared to healthy controls, n=10 (**D**, right). Individual, median, and interquartile range values are depicted; **p<0.01, Mann-Whitney U test.

Furthermore, we investigated inflammatory and redox biomarker changes at the end of the intensive phase of antituberculosis treatment. Two months after the initial visit (t2), we measured the levels of molecules denoting inflammation and redox state overall. Surprisingly, while the levels of pro-inflammatory eicosanoids only showed a reduction in LTB4 (**Figure 5A**), all the pro-resolutory lipids decreased significantly (**Figure 5B**). This outcome was unexpected, as we initially anticipated an increase in pro-resolutory lipids by the end of the intensive treatment phase. To gain more insights, we calculated the pro-resolutory/pro-inflammatory eicosanoid ratios and found that the LXA4/LTB4 ratio was the only one that increased after two months of treatment (**Figure 5C**, lower left). However, due to the persistently high levels of PGE2, all pro-resolutory lipids to PGE2 ratios were reduced. Other pro-inflammatory mediators, such as IL-8 and cell-free nucleosomes, remained unchanged after two months of treatment (t2) (**Figures 6A, B**), whereas TNF-α remained undetectable (data not shown). Moreover, all indicators of the prooxidant state showed significant reductions, while the total antioxidant level increased (**Figure 6C**). The SGRQ total score, which reflects the overall quality of life, showed a substantial reduction at t2 (**Figure 6D**, left). To better understand the extent of this reduction, we compared the patients’ scores with that of a group of ten healthy individuals who had recovered from COVID-19 without residual lung damage (**Figure 6D**, right). Despite continuous treatment, TB patients still experienced poorer quality of life.

### 3.5 Longitudinal dynamics of eicosanoids, oxidative stress biomarkers, and quality of life in tuberculosis patients

**Figure 7:**
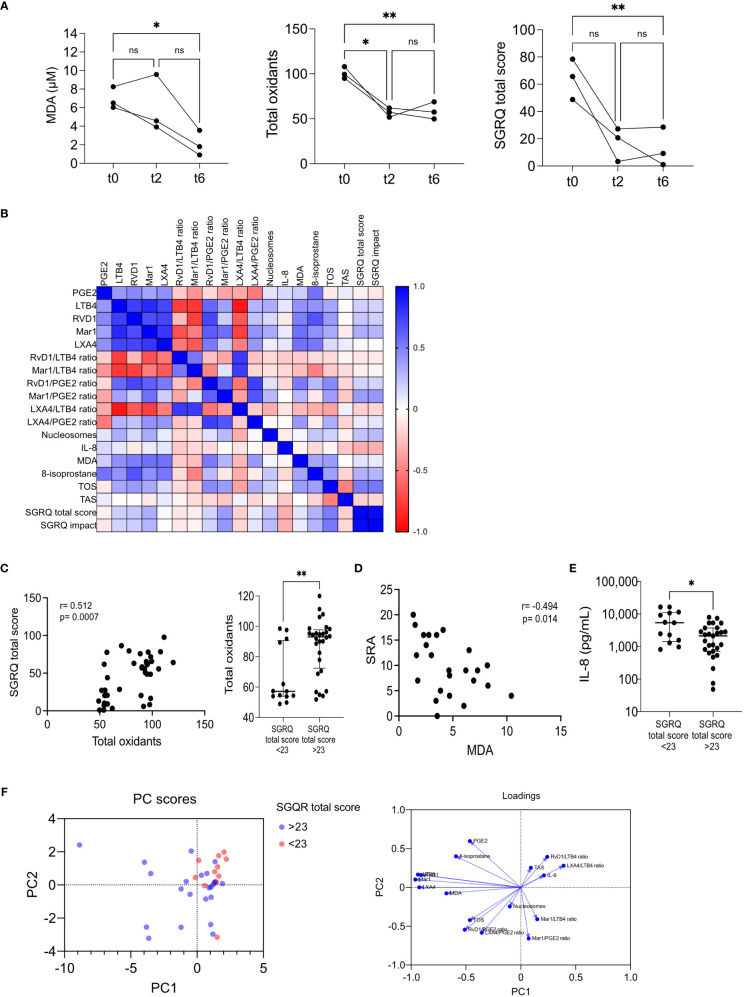
Modulation of all biomarkers in time. **(A)** MDA, total oxidants, and SGRQ total score at t0, t2, and t6, n=3; *p<0.05, **p<0.01, One-way ANOVA followed by Geisser-Greenhouse’s epsilon and Holm-Šídák’s multiple comparisons tests. **(B)** Spearman’s correlation matrix of eleven lipid mediator indicators, one tissue damage marker, one pro-inflammatory cytokine, four redox state indicators, and two QoL indicators, n=39; depicted is the heat map of Rho’s values. Total oxidants vs. SGRQ total score **(C)** and SRA vs. MDA **(D)** correlations; depicted are Spearman’s Rho graphs. Association of total oxidants (**C**, right) and IL-8 with QoL **(E)**. Individual values with medians and interquartile ranges are depicted, n=11; ns= not significant, *p<0.05, **p<0.01, Wilcoxon’s Rank sum. **(F)** Principal component analysis was performed; the PC scores plot depicted the subjects with SGRQ total scores higher (blue) or lower (red) than 23. The loadings plot showed the variables’ contribution to each component.

Three patients returned for a six-month follow-up (t6). We observed a continued decrease in eicosanoids and 8-isoprostane during this period, indicating reduced inflammation (**Supplementary Figure 3**). However, only one biomarker, MDA, along with total oxidants and the SGRQ total score, showed significant reductions (**Figure 7A**). We initially aimed to explore how circulating biomarkers correlated with the quality of life. Still, those associations could not be established due to a lack of patients with medium or low SGRQ scores at the initial visit. To gain a broader understanding, we created a correlation matrix including results from t0, t2, and t6 (**Figure 7B**). This analysis revealed strengthened correlations and similar patterns to our previous findings (**Figure 1A**, **Supplementary Material 1**). Notably, we found a significant correlation between the SGRQ total score and total oxidants, supporting the stratification of SGRQ scores (**Figure 7C**). An inverse correlation between SRA and MDA was also observed (**Figure 7D**). Furthermore, we confirmed that higher SGRQ scores were associated with lower levels of IL-8 (**Figure 7E**). Our findings highlight the importance of prioritizing total oxidants and lipoperoxidation indicators for further analysis as biomarkers in the management of TB, particularly in relation to quality-of-life outcomes.

We conducted a Principal Component Analysis (PCA) to examine potential connections between a set of biomarkers and QoL improvements. Patients with better QoL (defined by SGRQ total score <23) were represented by red markers, while blue markers represented those with worse QoL (SGRQ total score >23). Upon analyzing the PCA scores plot, we observed that the red markers predominantly clustered in the upper-right quadrant of the graph, where both PC1 and PC2 exhibited positive values. This clustering pattern in the upper-right quadrant suggests that lower SGRQ total scores might be linked to specific underlying factors contributing to positive values along both principal components (**Figure 7F**, left). The variables that most significantly contribute to the disparities observed in the PCA scores plot are visually presented in the loadings plot (**Figure 7F**, right). This loadings plot reveals that certain variables, such as IL-8, total antioxidants (TAS), LXA4/LTB4 ratio, and RvD1/LTB4 ratio, exhibit similar patterns along PC1 and PC2. These variables are associated with lower SGRQ scores. Conversely, variables like MDA, total oxidants (TOS), nucleosomes, RvD1/PGE2 ratio, and LXA4/PGE2 ratio display negative correlations with both PC1 and PC2, contributing to the patterns observed in patients with higher SGRQ scores.

## 4 Discussion

Numerous individuals affected by TB, including those with multidrug-resistant strains, experience long-term lung damage despite being deemed cured (5). The present study sought to identify potential serum biomarkers that could serve as early indicators of pulmonary dysfunction. No definitive biomarker has been clearly identified as predictive despite the known link between pulmonary impairment and increased inflammation and antioxidant system deficiencies (35, 36). Hence, additional investigations are necessary to identify suitable biomarkers that can accurately predict the development of lung dysfunction. Such predictive tools would modify patient management and care, substantially improving treatment outcomes.

We measured serum metabolites by ELISA because it is a convenient and easy-to-adopt technique. We included pro-inflammatory and pro-resolutory eicosanoids, pro-inflammatory cytokines (TNF-α and IL-8), cell-free nucleosomes, and indicators of the redox state as potential biomarkers. Furthermore, we incorporated indicators of disease severity and assessed the impact of respiratory distress symptoms on patients’ daily activities and psychosocial well-being through the St. George’s health-related QoL questionnaire (SGRQ). Pulmonary impairment resembles COPD and is highly associated with a poor QoL, hence the SGRQ total score correlates with pulmonary function (4, 37, 38).

During the initial stages of treatment, we observed limited associations between QoL indicators and lung damage, as well as other variables. However, our analysis did reveal significant correlations between scores obtained from the SGRQ and the Score of Radiologic Abnormalities (SRA), indicating a meaningful relationship between QoL measurements and lung damage. In exploring the potential links between serum metabolites and disease severity, our findings differed from previous studies that reported an association between inflammatory and prooxidant metabolites with tuberculosis severity. Surprisingly, we did not observe these associations, despite the anticipated increase in systemic low-grade inflammation and immune dysregulation associated to the high prevalence of diabetes among our patients (39). Moreover, we encountered three unexpected outcomes: notably low circulating levels of TNF-α, significantly lower IL-8 levels in patients with the poorest QoL, and considerably higher levels of total antioxidants in patients experiencing the worst QoL.

SGRQ has been recognized as a valuable tool for assessing the QoL in individuals who have recovered from TB (5). Based on our findings, measuring QoL indicators can help us understand the relationship between lung damage and the impact of respiratory symptoms on a patient’s life. To explore this further, we investigated the dynamics of QoL after treatment initiation. Previous studies have indicated that QoL scores tend to decrease over time, with the most significant improvements occurring during the intensive treatment phase (6, 40). Therefore, we focused our analysis on the metabolites two months after initiating treatment (t2).

Our observations revealed a significant decrease in the circulating levels of LTB4, RvD1, Mar1, and LXA4, but no reduction was observed in PGE2 at t2. The absence of a decline in PGE2 levels reduced the pro-resolutory to pro-inflammatory eicosanoid ratios. However, we continued to observe an increase in the LXA4/LTB4 ratio, as expected for a milder disease (22). Interestingly, the levels of pro-inflammatory mediators, namely TNF-α, IL-8, and cell-free nucleosomes, remained unchanged after two months of treatment. This finding was unexpected, as it was anticipated that TNF-α levels would decrease by the second month of therapy (41). It is worth noticing that the screening for neutrophil extracellular traps (NETs) as a component of lung damage is typically performed on airway-derived samples (42, 43), which may explain why fluctuations in these markers may not be reflected in the circulation.

In patients with pulmonary TB, SGRQ scores decreased significantly at t2 but remained substantially higher than healthy controls. These findings align with a previous study demonstrating a notable improvement in SGRQ total scores after four weeks of treatment; however, even at the six-month follow-up, patients showed evidence of residual pulmonary disability (44). It is worth mentioning that a median SGRQ total score below 30 has been linked to sputum conversion within the first month of treatment (45), and our patients fell within this range. In the subset of patients evaluated at six months of treatment (t6), the SGRQ total score and prooxidant biomarkers continued to decrease. Previous reports indicate that despite achieving “successful” treatment outcomes and reporting good quality of life, 27% of patients with TB still experience at least moderate to severe pulmonary function impairment, and 57% continue to exhibit respiratory symptoms after six months (44). Our findings reinforce the association between prooxidant metabolites and QoL, most likely reflecting the extent of pulmonary impairment.

This study provides valuable insights into longitudinal variations in eicosanoids, oxidative stress biomarkers, and QoL scores in TB patients throughout six months of treatment. By examining associations between circulating biomarkers and QoL and prioritizing critical variables based on their strength of association, we elucidated the complex interplay between biomarkers, oxidative stress, and QoL outcomes. These findings highlight the significance of total oxidants and lipoperoxidation indicators in TB management and emphasize the need to prioritize their analysis as biomarkers across different stages of the disease. Measuring QoL and redox state indicators can aid in monitoring treatment efficacy: an improvement in SGRQ scores during treatment may indicate a lower risk of failure, while worsening scores after successful treatment may suggest a higher risk for recurrence. Early identification of biomarkers indicating lung impairment in patients with TB is crucial for understanding long-term morbidities and designing interventions to optimize quality of life and productivity.

Our study has limitations, including the small sample size, the lack of follow-up of all patients, and the absence of respiratory tests. More exhaustive experiments are needed to validate these biomarkers. However, the Principal Component Analysis (PCA) outcomes delineated a discernible relationship between specific biomarkers and the QoL as gauged by the SGRQ scores. Specifically, IL-8, total antioxidants (TAS), LXA4/LTB4 ratio, and RvD1/LTB4 ratio exhibited a notable association with diminished SGRQ scores, indicative of an enhanced QoL. Conversely, patients with elevated SGRQ scores did not indicate a discernible pattern, thereby confounding the identification of a definitive biomarker ensemble linked to QoL deterioration. Nonetheless, the potential for monitoring QoL enhancements can be inferred from IL-8, total antioxidants (TAS), LXA4/LTB4 ratio, and RvD1/LTB4 ratio. These ratios collectively signify a prominence of pro-resolutive eicosanoids over pro-inflammatory counterparts, thereby corroborating the amelioration of QoL. This analytical insight thus underscores the utility of the biomarkers as potential indicators of QoL progression that could be used for disease and treatment monitoring.

## Data availability statement

The original contributions presented in the study are included in the article/**Supplementary Material**. Further inquiries can be directed to the corresponding author.

## Ethics statement

The studies involving humans were approved by Institutional Review Board from the Instituto Nacional de Enfermedades Respiratorias Ismael Cosío Villegas. The studies were conducted in accordance with the local legislation and institutional requirements. The participants provided their written informed consent to participate in this study.

## Author contributions

EJ and LC-B contributed to the conception and design of the study. MM-T, LC-B, and LS-M collected the samples and organized the database. EJ, LS-M, SG-B, and CC performed the experiments. EJ, MT, and YG analysed the data. EJ and LC-B wrote the first draft of the manuscript. MT and YG revised the manuscript critically for important intellectual content. All authors contributed to the manuscript revision and read and approved the submitted version.
