# Supplementary figures and images for: Serum pro-inflammatory biomarkers associated with improvement in quality of life in pulmonary tuberculosis
Source: Front Immunol. 2023 Sep 11;14:1241121. doi: 10.3389/fimmu.2023.1241121 (PMC10518397; doi:10.3389/fimmu.2023.1241121)

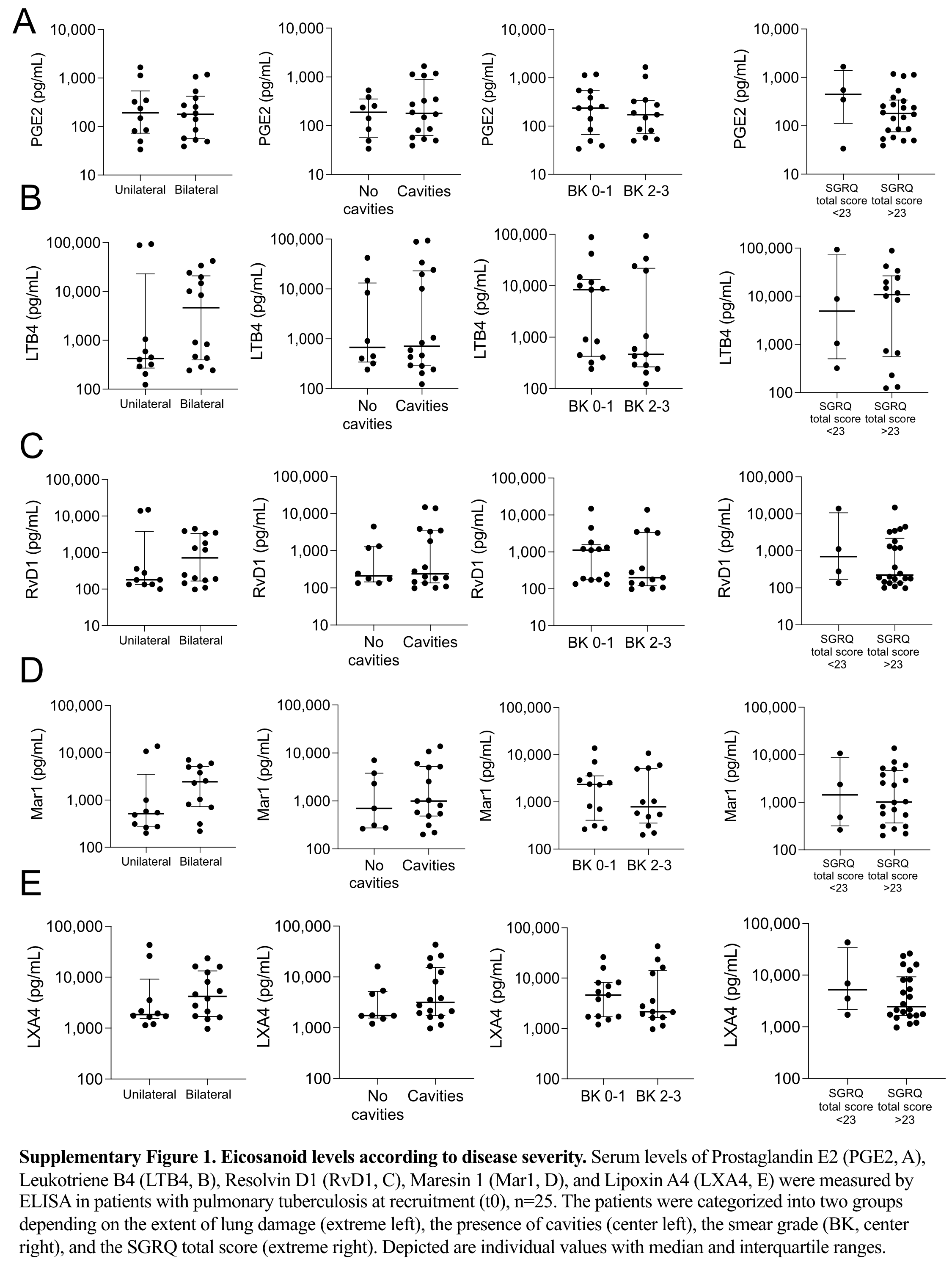

Supplement: Supplementary file 1 [file Image_1.tiff]

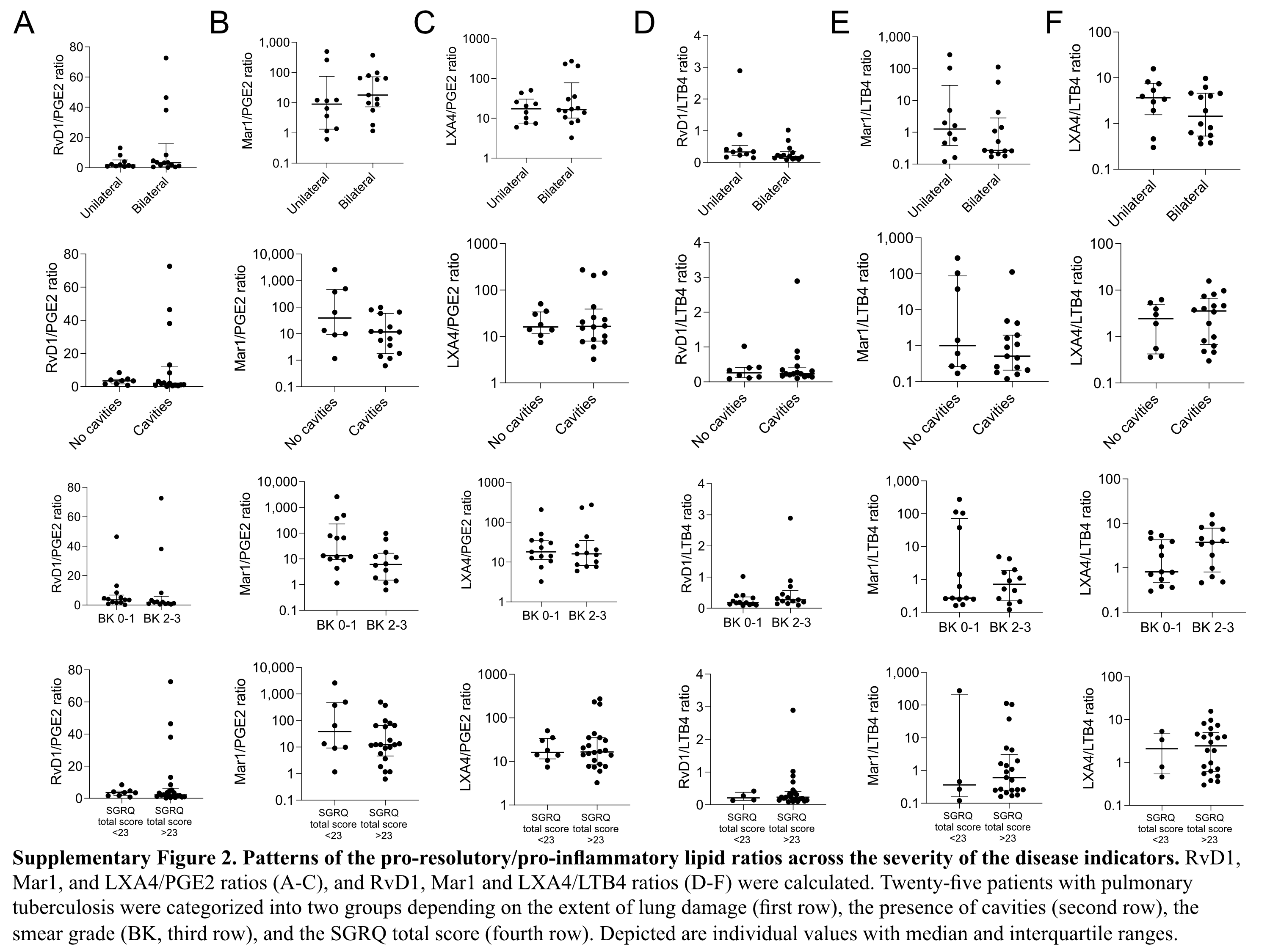

Supplement: Supplementary file 2 [file Image_2.tiff]

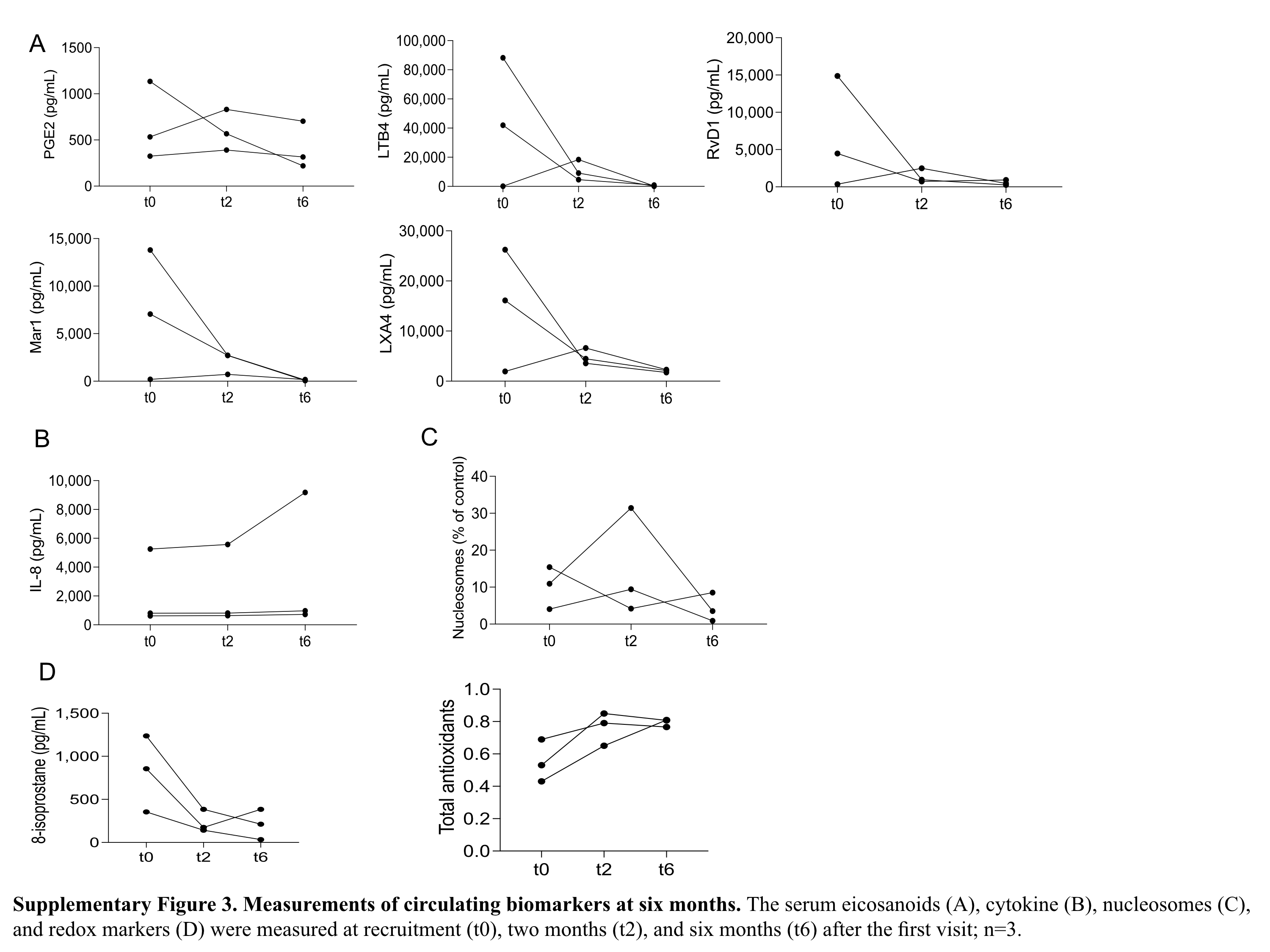

Supplement: Supplementary file 3 [file Image_3.tiff]
